# Comparative sentiment and thematic analysis of depression-related discourse between healthcare professionals and non-healthcare individuals on Chinese social media: a computational text analysis study

**DOI:** 10.3389/fpsyg.2026.1858436

**Published:** 2026-07-28

**Authors:** Qi Zhou, Yuxi Chen, Yi Li, Lili Yang, Qinrou Yu, Lihui Yan, Danhong Qian

**Affiliations:** 1 Hangzhou Linping District Integrated Traditional Chinese and Western Medicine Hospital, Hangzhou, China; 2 Medicine department, Huzhou University, Hangzhou, Zhejiang, China; 3 Nursing education department, Sir Run Run Shaw Hospital of Zhejiang University, Hangzhou, China; 4 Nursing department, Hangzhou Normal University, Hangzhou, Zhejiang, China

**Keywords:** depression, K-means algorithm, nursing, sentiment analysis, social media

## Abstract

**Objective:**

This study analyzes differences in depression-related expressions between healthcare professionals and non-healthcare individuals on Chinese social media.

**Background:**

Sentiment plays a critical role in depression management, influencing condition assessment, social support, and treatment adherence. However, traditional research methods are limited by self-report bias, small samples, and constrained expression. Social media offers a unique platform to capture authentic emotions at scale.

**Methods:**

A total of 120,895 comments were retrieved from Zhihu, Weibo, and Xiaohongshu. Data underwent cleaning, relevance verification, and anonymization. Users were classified into healthcare professionals and non-healthcare individuals through profile verification and manual annotation. Sentiment analysis applied the Dalian University of Technology Sentiment Lexicon, adapted to Chinese contexts and grounded in Ekman's emotional framework (anger, disgust, fear, sadness, surprise, positive emotion). Polarity and intensity (−1 to 1) were quantified. Content analysis employed a K-Means clustering algorithm to identify thematic patterns. Content analysis was conducted using computational text analysis methods, including sentiment analysis and K-means clustering, to identify emotional and thematic patterns in depression-related social media discourse.

**Results:**

Non-healthcare individuals expressed a wider emotional spectrum (anger, disgust, fear, sadness, surprise, and positive emotions), while healthcare professionals primarily expressed anger, fear, and positive emotions. Non-healthcare discourse focused on illness experiences, stigma, and treatment conflict, whereas healthcare professionals emphasized occupational stress, emotional labor, and professional fulfillment.

**Conclusions:**

The results highlight divergent emotional orientations: patients and the public center on illness experiences, while professionals emphasize identity and occupational strain. These differences underscore the need for tailored communication strategies, empathy training, and institutional psychological support for healthcare providers.

## 1 Background

Depression is a prevalent mental disorder defined by symptoms including persistent low mood, diminished interest, and social withdrawal, which severely affects patients' daily life and social functioning (Snaith, 1987). The global incidence of depression is increasing and has emerged as a leading causes of the global burden (Liu et al., 2024). According to the World Health Organization, approximately 1 billion people worldwide suffer from mental disorders, with around 95 million individuals in China experiencing depressive disorders. The global burden of mental health issues has intensified following the COVID-19 pandemic, the number of people suffering from depression surged by 53 million, marking a 27.6% increase (The 2022 National Mental Health Survey Report, 2023). More seriously, depression has become an important risk factor for suicide (Wang et al., 2025; Deska et al., 2025; Cheng et al., 2024; Sharma and Sharma, 2021). Emotional expressions of depressed patients usually show negative tendencies, such as sadness, helplessness, and despair (Fornaro et al., 2025; García-Fernández et al., 2025). Research indicates that the sentiment of patients with depression is closely linked to the their condition assessment and directly influences social support and treatment adherence (Zaid et al., 2025; Arpaci and Tanriverdi, 2024). Therefore, it is of great practical significance to study the sentiment of depressed patients (Arpaci and Tanriverdi, 2024). Analyzing the sentiment of depressed patients helps healthcare professionals and non-healthcare in comprehending the internal experiences of the depressed, thereby facilitating effective interventions.

Sentiment analysis of depressed patients relies on traditional methods and natural language processing (NLP) methods. Traditional methods, including clinical interviews and questionnaires, rely significantly on patients' self-reported data in specific contexts. Healthcare professionals typically use the Patient Health Questionnaire (PHQ-9) scale to assess depressed emotion (Kroenke et al., 2001), along with clinical interviews to analyze patients' expression (Chen et al., 2025). Sentiment Analysis (SA) is a method of text analysis that uses Natural Language Processing (NLP) and machine learning, which is widely used in the fields of mental health, market analysis and social opinion monitoring (Helmy et al., 2024; Nor et al., 2022).

Traditional research methods are constrained by limited data sources, regulated expression, and challenges in capturing authentic emotions. First, the traditional method is subject to social desirability bias. Respondents may adjust their responses due to Social Desirability Bias or self-protective tendencies to avoid being labeled as “mentally ill,” resulting in compromised authenticity of the research data (Nawaz et al., 2025). Secondly, traditional methods have the problem of limited data. Traditional studies rely on clinical data sourced from hospitals or mental health organizations, which primarily include patients who have received a diagnosis or are inclined to seek help. However, many potential patients remain outside the healthcare system, making their emotional experiences are difficult to capture. Third, traditional methods are hindered by the controlled respondent expression (Zhang et al., 2024). In formal interviews or questionnaire completion, patients are contextually constrained, making it difficult for them to spontaneously express their emotions and therefore potentially underestimating their true negative feelings. Patients with depression often struggle to express negative emotions in face-to-face interactions, favoring the expression of their true feelings in anonymous environments.

NLP methods have the limitation in few applications and lacking intrinsic psychological factors. Existing depression studies relying on natural language methods for sentiment analysis are based on English texts such as Twitter and Reddit (Obagbuwa et al., 2023; Qasim et al., 2025) and lack a Chinese perspective. In addition, NLP methods are limited to identifying positive and negative emotions, lacking intrinsic analyses of the underlying causes of sentiment scores (Zhang et al., 2024; Jakate et al., 2023; Jung et al., 2017). Therefore, existing studies lack a private perspective to capture the real emotional expressions of Chinese depression patients and the underlying psychological factors.

In recent years, social media has become an important platform for information dissemination and opinion exchange. Compared with traditional offline interviews, social media provide a open, anonymous, and interactive communication environment, enabling users to express their emotions, share experiences, and seek support freely (Bucur et al., 2025). For patients with depression, social media serves as a significant medium for knowledge acquisition and a major place for emotional catharsis (Yan et al., 2025). In an anonymous or semi-anonymous environment, patients can express their real emotions without fear of social stigma. However, depression discussions on social media are often hindered by misconceptions, stereotypes, and cultural conflicts (Sun and Fang, 2024). The public still perceive depression as indicative of “pretentiousness” or “weakness of will,” and these negative perceptions may exacerbate the psychological burden experienced by patients (Zhang et al., 2024). Moreover, differences in the perception and acceptance of depression across socio-cultural contexts can result in biased information dissemination. This study aims to analyze the attitudes of healthcare professionals and non-healthcare toward depression using social media, addressing the limitations of traditional methods and providing a realistic socio-emotional landscape.

Although previous studies have explored depression-related sentiment on social media, most focused primarily on general public discourse or binary positive–negative emotional classification, with limited attention to differences between healthcare professionals and non-healthcare individuals. Healthcare professionals possess clinical knowledge and professional experience in depression management, whereas non-healthcare individuals are more likely to express illness experiences, stigma perceptions, and treatment misunderstandings from a public perspective. Comparing these two groups may help reveal the cognitive and emotional gap between professional medical understanding and public perceptions of depression. Such differences are important for improving health communication strategies, reducing misinformation and stigma, and developing targeted mental health interventions on Chinese social media platforms.

## 2 Methods

### 2.1 Data collection

Data were gathered using “depression” as the primary search term across Zhihu, Weibo, and Xiaohongshu, covering entries up to September 1, 2024. The data collection period was predefined according to the study design and aimed to establish a consistent analytical dataset for comparing emotional expression patterns between healthcare professionals and non-healthcare individuals. Although social media discourse may change over time, this study focused on identifying differences in emotional and thematic characteristics within a specific observation period rather than conducting continuous real-time monitoring. Extracted variables included date, user type, text content, and engagement metrics. Users were classified into healthcare professionals, non-healthcare individuals, media organizations, and unknown. Healthcare professionals were identified through profile metadata (e.g., hospital affiliation, verified accounts) and cross-checked by manual annotation (King et al., 2024). Non-healthcare individuals were further categorized into self, family/friend, celebrity, and media organizations. Discrepancies were resolved by double coders with kappa > 0.85.

### 2.2 Data cleaning

Data cleaning consisted of both automated preprocessing and manual verification. During automated preprocessing, exact duplicate records were identified using unique text matching and removed. Commercial advertisements, promotional messages, spam content, posts containing only hyperlinks or emojis, and empty or extremely short comments (fewer than five Chinese characters after preprocessing) were automatically excluded using rule-based filtering. Automatic language detection was then applied to retain only Chinese- and English-language posts. Subsequently, two trained researchers independently reviewed posts that could not be confidently classified by the automated procedures, particularly comments with ambiguous semantic content or uncertain relevance to depression-related discussions. Posts judged to be unrelated to depression (e.g., metaphorical use of “depression,” entertainment-related expressions, or irrelevant emotional discussions) were excluded following consensus between reviewers. Text preprocessing further included punctuation removal, spelling correction, synonym normalization, and anonymization of identifiable information such as usernames, telephone numbers, and email addresses.

Users were classified into healthcare professionals, non-healthcare individuals, media organizations, and unknown users according to predefined criteria. However, the primary comparative analyses focused on healthcare professionals and non-healthcare individuals because the study aimed to compare emotional expressions between individual users rather than organizational communication. Users were first classified into four mutually exclusive categories: healthcare professionals, non-healthcare individuals, media organizations, and unknown users. Healthcare professionals were identified through verified occupational information and manual review. Media organizations comprised official institutional or news accounts. Unknown users lacked sufficient profile information for reliable classification. Descriptive subcategories within non-healthcare individuals (e.g., self, family/friends, celebrities) were used only for descriptive purposes and were not treated as separate analytical groups.

### 2.3 Relevance verification

Two independent evaluators reviewed samples to confirm relevance to depression discourse. Ambiguous cases were adjudicated by senior researchers. This rigorous filtering ensured validity of user group distinctions.

### 2.4 Sentiment analysis

Sentiment analysis was performed using the Dalian University of Technology Sentiment Lexicon, which has been applied in previous Chinese text-mining studies (Cero et al., 2024; Yazdani et al., 2023; Zhou et al., 2024). This method was selected because it provides interpretable emotional categories and allows large-scale analysis of social media texts. Chinese word segmentation was conducted using Jieba, and synonym normalization was performed using HOWNET. Negation and antonym rules were incorporated to improve emotional classification.

Emotional categories were classified according to Ekman's basic emotion framework, including anger, disgust, fear, sadness, surprise, and positive emotion. Emotional polarity was quantified on a scale ranging from −1 to +1, and multi-label classification was applied because individual comments could contain multiple emotional expressions.

### 2.5 Content analysis using the K-means algorithm

Content analysis was conducted using the K-means clustering algorithm to identify thematic patterns in depression-related social media discourse. Each social media comment was treated as an independent textual unit. After text preprocessing and Chinese word segmentation using Jieba, each comment was transformed into a numerical feature vector using the Term Frequency–Inverse Document Frequency (TF-IDF) representation. TF-IDF weighting was adopted because it effectively captures the relative importance of words while reducing the influence of frequently occurring but less informative terms, making it suitable for unsupervised clustering of large-scale social media text. The resulting TF-IDF matrix served as the input for subsequent K-means clustering.

Each comment was converted into a TF-IDF feature vector before clustering. K-means clustering was implemented using Python (version 3.11) and the scikit-learn package (version 1.5; Zhou et al., 2023). The optimal number of clusters was determined using the elbow method based on within-cluster sum of squares (WCSS). The algorithm employed k-means++ initialization with 20 random initializations (n_init = 20), a maximum of 300 iterations (max_iter = 300), a convergence tolerance of 1 × 10^−4^, and a fixed random seed (random_state = 42) to improve reproducibility. Following clustering, representative comments and high-frequency keywords from each cluster were manually reviewed by two independent researchers to verify semantic coherence before assigning thematic labels.

### 2.6 Ethics approval

This study analyzed publicly available comments from openaccess Chinese social media platforms and did not involve direct interaction with users or the collection of private or identifiable personal information. During data preprocessing, all usernames and other potentially identifiable information were removed through anonymization before analysis. The study protocol was reviewed and approved by the Institutional Review Board of Hangzhou Linping District Integrated Traditional Chinese and Western Medicine Hospital (Approval No.: 2025_L_006). All procedures complied with relevant institutional guidelines and ethical principles for research involving publicly available online data.

## 3 Results

This study included 120,895 comments, totaling 3,419,299 words. The results of user type showed that Non-healthcare is 84.6%, health professional (6.5%), media organization (6.5%), and unknown (2.4%).·Non-healthcare individuals (84.6%): Six emotions identified—anger, disgust, fear, sadness, surprise, positive emotion. Themes included treatment adherence, stigma, suicidal ideation, and cognitive conflict. Healthcare professionals (6.5%): Expressed primarily anger, fear, and positive emotions. Themes included burnout, emotional labor, and professional fulfillment. Other groups: Media organizations (6.5%) and unknown users (2.4%) were excluded from the main analysis but noted as potential future comparison groups.

The identified emotional and thematic patterns represent differences in depression-related discourse characteristics between healthcare professionals and non-healthcare individuals within the analyzed social media dataset.

Non-healthcare individuals expressed a wider emotional spectrum (anger, disgust, fear, sadness, surprise, and positive emotions), while healthcare professionals primarily expressed anger, fear, and positive emotions. Non-healthcare discourse focused on illness experiences, stigma, and treatment conflict, whereas healthcare professionals emphasized occupational stress, emotional labor, and professional fulfillment.

Non-healthcare have 6 emotions about depression (Table 1). Good emotions include gratitude and respect, treatment adherence, and humanistic medicine. Surprise emotions include cognitive bias, understanding differences, and cognitive conflict over treatment. Anger emotions include trust crisis, loss of partial decision-making autonomy, and misunderstanding. Fear emotions include irreversible changes and stigma exposure. Disgust emotions include stigma internalization and public prejudice. Sad emotions includes suicidal ideation and emotional-physiological interactions.

**Table 1:** Sentiment analysis and content analysis of non-healthcare.

Emotion	Related sentiment analysis word	Content analysis
Good	Appreciation, recognition, admiration, thankfulness, respect	Gratitude and respect
	Awareness, curiosity, open-mindedness, recognition, acceptance	Treatment adherence
	Compassion, empathy, warmth, understanding, support	Humanistic medicine
Surprise	Misconception, shock, skepticism, realization, reevaluation	Cognitive bias
	Awareness, curiosity, open-mindedness, recognition, acceptance	Understanding differences
	Dissonance, confusion, hesitation, uncertainty, reassessment	Cognitive conflict in treatment
Anger	Betrayal, disappointment, distrust, resentment, frustration	Trust crisis
	Helplessness, powerlessness, rebellion, resistance, annoyance	Loss of partial decision-making autonomy
	Irritation, alienation, indignation, exasperation, agitation	Misunderstanding
Fear	Anxiety, panic, helplessness, hopelessness, dread	Irreversible Changes
	Shame, embarrassment, vulnerability, rejection, distress	Stigma exposure
Sad	Fatigue, weakness, despair, melancholy, suffering	Emotional-physiological interactions
	Hopelessness, isolation, worthlessness, pain, desperation	Suicidal ideation
Disgust	Aversion, contempt, frustration, outrage, resentment	Public prejudice
	Self-loathing, shame, inferiority, guilt, disdain	Stigma internalization

Health professionals have 3 emotions about depression (Table 2). Good emotions include emotional labor and professional fulfillment. Fear emotions include emotional management in high-risk scenarios and occupational psychological support systems. Anger emotions include burnout and conflict between patient and expertise.

**Table 2:** Sentiment analysis and content analysis of healthcare professional.

Emotion	Related sentiment analysis word	Content analysis
Good	Responsibility, adaptability, emotional investment, self-mastery, perseverance	Emotional labor
	Accomplishment, self-worth, motivation, recognition, career growth	Professional fulfillment
Anger	Frustration, depletion, impatience, weariness, resignation	Burnout
	Disagreement, tension, resistance, irritation, power imbalance	Conflict between patient and expertise
Fear	Caution, hyper-awareness, mental strain, decision anxiety, readiness pressure	Emotional management in high-risk scenarios
	Emotional dependency, reassurance seeking, psychological burden, support-seeking, mental fatigue	Occupational psychological support

## 4 Discussion

These findings should be interpreted as differences in online emotional expression and thematic construction rather than direct evidence of differences in psychological states between the healthcare professionals and non-healthcare individuals. Non-healthcare participants primarily expressed illness-centered emotions, including stigma exposure, suicidal ideation, cognitive conflict regarding treatment, and fear of irreversible personal changes. In contrast, healthcare professionals expressed occupation-centered emotions, particularly emotional labor, burnout, communication pressure, and professional fulfillment. These findings suggest that healthcare professionals tend to interpret depression through the lens of clinical responsibility and occupational stress, whereas non-healthcare individuals are more likely to frame depression through lived emotional experiences and social stigma. This divergence reflects a broader knowledge and perception gap between professional medical discourse and public understanding of depression. While healthcare professionals emphasize treatment adherence, emotional management, and clinical communication, non-healthcare individuals frequently express uncertainty, misunderstanding, and distrust regarding depression treatment. The findings therefore indicate the necessity of differentiated communication strategies. Public-oriented interventions should prioritize psychoeducation, stigma reduction, and accessible mental health communication, whereas interventions targeting healthcare professionals should focus on emotional resilience, burnout reduction, and occupational psychological support systems.

### 4.1 Sentiment analysis of non-healthcare

Good emotion of non-healthcare include gratitude and respect, treatment adherence, and humanistic medicine. Firstly, Gratitude and respect reflect the patient's acknowledgment of medical interventions. This outcome may be associated with the physician's empathy capacity, the healthcare team support, and the patient's recovery perception. The results are consistent with the study of Cheng et al. (2015). Secondly, treatment adherence indicates patients' confidence in physicians' recommendations. This outcome may be associated with the clarity of medical information, physicians communication, and the patients' health literacy. The results are consistent with the study of Meng et al. (2024). Treatment adherence is not merely a one-way execution of medical recommendations; it is influenced by patients' identification with their physicians and their enhanced sense. Patients may experience security from regular follow-ups and medication routines, which can enhance emotional stability. Finally, Humanistic therapies reflect patient recognition of the person-centered model of care. The results are consistent with the study of Hunot et al. (2013) and Shinohara et al. (2013). This result reflects the value of emotional support in alleviating medical anxiety. Some patients may experience compensatory emotional comfort within the medical environment as a result of chronic social support deficits. This psychological compensatory effect may increase motivation for treatment and promote a greater willingness to express positive emotions. Therefore, this study proposes that hospital administrators strengthen communication training for healthcare professionals and improve empathy so that more patients can feel humanistic care. Secondly, the delivery of medical information must be optimized to enhance patient comprehension of the treatment plan, thereby increasing compliance.

Surprise emotion of non-healthcare include cognitive bias, understanding differences, and cognitive conflict in treatment. Firstly, cognitive bias may be associated with patients' misconceptions regarding depression. The results are consistent with the study of Everaert et al. (2012). Some patients may be surprised by the physiological mechanisms underlying depression upon diagnosis. They may attribute it to character flaws or external stresses rather than physiological factors. This result suggests that societal views on depression continue to be shaped by the stigma associated with mental illness, leading to an underappreciation of its medical aspects. Secondly, understanding differences may stem from the complexity of the doctor's professional terminology, which may be challenging for the patient to grasp, or from the patient's inability to fully process the information in a high-pressure context. The results are consistent with the study of Sithambaram et al. (2024). Finally, the results of cognitive conflict in treatments indicate that patients may favor non-pharmacological interventions, including psychotherapy and exercise therapy. Patients viewed the use of antidepressants as a serious illness or losing self-control. This behavior contradicts their perception of the severity of their psychological issues. The results are consistent with the study of Feixas et al. (2014). Therefore, hospital administrators should develop accessible health education materials on depression that are tailored to the cultural context and cognitive abilities. Training sessions will be organized to improve doctors' ability to communicate the subject and reduce communication barriers.

Anger emotion of non-healthcare include trust crisis, loss of partial decision-making autonomy, and misunderstanding. The results of trust crisis indicate that patients express dissatisfaction with treatment outcomes, particularly in the absence of short-term improvements. The results are consistent with the study of Lai et al. (2023) and Imai et al. (2020). Therefore, physicians must have the capability to assess emotions for early detection and recognize potential medical conflicts. Second, loss of partial decision-making autonomy suggests that family members or physicians making treatment decisions without the patient's complete comprehension. The experience of passive treatment acceptance may result in anger and potential dissatisfaction with prior decision-making processes. The results are consistent with the study of Simon et al. (2007). Finally, the results of misunderstanding highlight the insufficient intuitive physiological representations of depression symptoms relative to other physical illnesses, resulting in challenges patients encounter in both work and home settings. This external denial may cause patients to perceive that their suffering is not being addressed. The results are consistent with the study of Zhang et al. (2024). Therefore, physicians must consider depressed patients' perceptions of decision-making authority and enhance patient involvement in medical decision-making processes. The treatment process increases the clarity of communication, thereby enhancing the patient's right to information.

Disgust emotion of non-healthcare include public prejudice and stigma internalization. Firstly, the results of stigma internalization indicate that patients are influenced by the society culture. The results are consistent with the study of Bouvet and Bouchoux (2015). People with depression may experience the process of double stigmatization. They perceive societal prejudices surrounding depression while simultaneously internalizing these biases, leading them to conclude that they are guilty. This self-stigmatization may lead to avoidance of treatment and a negative perception to their situation. Second, the results of public prejudice against mental illness suggest that media stereotypes and discriminatory socio-cultural discourses affect patients' self-esteem and willingness to seek treatment. The results are consistent with the study of Mickelson (2001). Therefore, physicians systematically assess the level of self-stigmatization in patients with depression during initial interviews or follow-up assessments. Secondly, physicians integrate anti-stigma interventions into psychotherapy to address patients' negative beliefs. Finally, doctors engage in community activities and invite recovered patients to share their experiences to reduce social misconceptions about depression.

Fear emotion of non-healthcare include irreversible changes and stigma exposure. First, the results of the fear of irreversible change suggest that patients may be concerned that depression could result in permanent brain damage or that the use of antidepressant medication might lose themself. This fear of change in self-identity may strengthen their opposition to treatment. The results are consistent with the study of O'Connor et al. (2002) and Gumuchian et al. (2024). Second, the result of stigma exposure suggests that seeking treatment may lead to being labeled by family, colleagues, or society as individuals with mental health issues. This societal fear may lead them to delay seeking treatment or even avoid discussing their condition openly. The results are consistent with the study of Oliver (2020). Therefore, this study suggests a stepped dosing strategy and real-time tolerance assessment for patients concerned about medication side effects, aiming to improve their confidence in treatment. Secondly, psychiatric outpatient clinics and counseling rooms should implement anonymous counseling channels to enable patients to seek professional advice without disclosing their identity, thereby mitigating the fear associated with the illness stigma.

Sad emotion of non-healthcare include emotional-physiological interactions and suicidal ideation. Firstly, the results of the emotional-physiological interaction suggest that patients may prefer to sustain feelings of sadness, as this can elicit external attention or serve as a means of expressing their needs. This phenomenon is particularly evident in individuals experiencing chronic deficits in social support. The results are consistent with the study of Millgram et al. (2015). Second, the results of suicidal ideation suggest that the risk of suicide may increase in depressed individuals during the early stages of remission. They start to regain mobility while their mood continues to be low. Mood alterations during this phase necessitate careful consideration. The results are consistent with the study of Wang et al. (2025), Deska et al. (2025), Cheng et al. (2024), and Sharma and Sharma (2021). Therefore, it is advisable for physicians to integrate psychosocial interventions for patients experiencing chronic sadness and insufficient social support, including family therapy or support groups, to improve their social connections. Secondly, physicians should enhance the monitoring of patients during the early phase of remission, particularly concentrating on the high-risk period characterized by “recovering mobility but still experiencing depression,” and modifying medication or increasing the frequency of psychological interventions as needed.

### 4.2 Sentiment analysis of health professionals

Good emotion of health professionals include emotional labor and professional fulfillment. Firstly, the result of emotional labor suggests that physicians must engage significantly in emotional labor when dealing with depressed patients, potentially leading to positive outcomes or considerable burdens. The results are consistent with the study of Kim et al. (2021) and Yom et al. (2017). Second, the result of professional fulfillment suggests that physicians derive professional value when they successfully help their patients. The results are consistent with the study of Cheng et al. (2015). Therefore, future research explores psychological recovery mechanisms for physicians after emotional labor, including mental toughness training and peer support groups, to reduce the possible psychological drain of long-term emotional engagement.

Anger emotion of health professionals include burnout and conflict between patient and expertise. Firstly, the results of burnout suggest that physicians may develop anger emotion due to prolonged high-pressure work and lacking understanding. The results are consistent with the study of Ryan et al. (2023). Secondly, the results of conflict between conflict between patient and expertise suggests that the disparity between health professionals' judgments and patients' understanding, potentially resulting in communication barriers. The results are consistent with the study of Sithambaram et al. (2024). Therefore, the hospital proposed a training about doctor-patient communication to evaluate the effectiveness of various communication techniques, such as empathic communication and motivational interviewing, in mitigating patients' misunderstandings and alleviating doctors' burnout.

Fear emotion of health professionals include emotional management in high-risk scenarios and occupational psychological support. Firstly, the results of emotional management in high-risk scenarios suggest that physicians need additional psychological adjustment skills when dealing with depressed patients with life-threatening conditions. The results are consistent with the study of Wang et al. (2012). Second, the results of the construction of occupational psychological support systems suggest that physicians may not receive sufficient support in managing patients' emotions, which affects occupational wellbeing. The results are consistent with the study of Li et al. (2018). Therefore, hospitals should establish a psychological support system for physicians, including the implementation of a psychological supervision mechanism and conducting emotional management training, to mitigate the effects of high-stress environments on physicians' mental wellbeing.

### 4.3 Implications

The comparative emotional patterns identified in this study provide important implications for mental health intervention, digital health communication, and public policy development. Non-healthcare individuals predominantly expressed stigma-related emotions, suicidal ideation, treatment conflict, distrust, and fear associated with depression, suggesting that misconceptions and culturally embedded stereotypes regarding mental illness remain highly prevalent in Chinese social media discourse. These emotional expressions indicate the urgent need for accessible psychoeducation, improved depression literacy, and anti-stigma interventions targeting the general public. Chinese social media platforms may therefore serve as important channels for culturally adaptive mental health education and public awareness campaigns.

In contrast, healthcare professionals primarily expressed emotions associated with emotional labor, occupational stress, burnout, and psychological burden in high-risk clinical scenarios. These findings highlight that healthcare professionals not only function as treatment providers, but are also emotionally vulnerable populations within depression-related care environments. Healthcare institutions should therefore establish structured occupational psychological support systems, including emotional resilience training, peer-support programs, communication skills training, and burnout prevention interventions to improve healthcare professionals' psychological wellbeing and communication capacity.

Furthermore, the significant emotional and cognitive differences observed between healthcare professionals and non-healthcare individuals reveal an important knowledge and communication gap between professional medical discourse and public perceptions of depression. Public-oriented communication strategies should focus on correcting misinformation, clarifying misconceptions surrounding antidepressant treatment, and reducing social discrimination against individuals with depression. Meanwhile, professional-oriented communication should emphasize empathy-based communication and emotional management in clinical practice. Additionally, the findings suggest that sentiment analysis of social media discourse may serve as a valuable tool for digital public health surveillance. Monitoring emotional trends, stigma-related narratives, and misinformation on social platforms may help policymakers and healthcare organizations dynamically identify emerging mental health concerns and optimize evidence-based health communication strategies. Therefore, this study contributes not only to understanding depression-related emotional expression, but also to the development of precision mental health communication frameworks and evidence-based digital mental health policies in China.

### 4.4 Limitations

There are two limitations to this study. First, health professionals and media organizations represent a small part in the internet. Therefore, we recommend that future studies add medical forum and news data to supplement the results. Second, the Non-healthcare represent a large younger demographic but lacks an elderly demographic. Older persons are the main target population, but social media overlook their voices. Future studies should focus on older persons' attitudes using qualitative study. Third, the proportions of healthcare professionals and non-healthcare individuals differed substantially because the dataset reflected naturally occurring participation patterns on social media platforms. Therefore, differences identified between groups should be interpreted as variations in online discourse characteristics rather than direct comparisons of population-level emotional experiences. Forth, social media discourse is dynamic and influenced by temporal changes, the findings should be interpreted within the specific period examined in this study. Future studies using longitudinal or continuously updated datasets may further explore changes in depression-related online emotional expression over time. Finally, although lexicon-based sentiment analysis provides interpretability and reproducibility, it may not fully capture complex contextual expressions such as sarcasm, metaphor, and implicit emotional meanings. Future research may incorporate advanced transformer-based language models to improve contextual emotion recognition. Although K-means provides an efficient and interpretable framework for large-scale text clustering, the present study did not perform algorithmic cross-validation using alternative topic modeling methods. Future studies should compare K-means with LDA, BERTopic, semantic network analysis, and transformer-based language models to evaluate the robustness of topic identification. Future studies should incorporate specialized depression communities, professional medical forums, and media organizations to further investigate differences in depression-related discourse across online environments. In addition, mainstream social media users are predominantly younger adults; therefore, future research should include older populations using complementary qualitative or clinical approaches.

## 5 Conclusions

This study indicates that the sentiment of non-healthcare personnel primarily focus on the illness experience, while health professionals focus on occupational stress and professional identity. This difference suggests that health professionals need to optimize doctor-patient communication, enhance health education, and improve treatment adherence, and address the emotional burden and provide a psychological support.

## Data Availability

The raw data supporting the conclusions of this article will be made available by the authors, without undue reservation.
