# Supplementary material for: Comparative sentiment and thematic analysis of depression-related discourse between healthcare professionals and non-healthcare individuals on Chinese social media: a computational text analysis study
Source: Front Psychol. 2026 Jul 28;17:1858436. doi: 10.3389/fpsyg.2026.1858436 (PMC13457727; doi:10.3389/fpsyg.2026.1858436)
Supplement: Supplementary file 1 [file Table_1.DOCX]

**Guidance for publishing qualitative research in informatics**

[Guidance for publishing qualitative research in informatics | EQUATOR Network (equator-network.org)](https://www.equator-network.org/reporting-guidelines/guidance-for-publishing-qualitative-research-in-informatics/)

| **Theory** |  |
| --- | --- |
| Cite theory appropriate to the topic being studied if applicable | NO |
| **Research question and study design** |  |
| a. State the research question b. State the study design and methodological perspective of the research | Yes |
|  | Yes |
| **Sampling** |  |
| a. Describe the sampling approach b. Describe any approaches to ensure the inclusion of people from marginalized or underserved groups c. Report and justify the sample size d. If using saturation to determine sample size, report what type of saturation was used, and how saturation was assessed* | Yes |
|  | Yes |
|  | Yes |
|  | Yes |
| **Data collection** |  |
| a. Report how data were collected b. Report any methods for reducing bias in data collection and analysis* | Yes |
|  | Yes |
| **Data analysis** |  |
| a. Describe data analysis methods, with appropriate citations* 1．For deductive analysis, report how the theory was used in the data collection and analysis* 2．For inductive analysis, report how the steps of inductive analysis were done* 3．For theory development, report how categories were developed* b. Describe any methods for improving the dependability of coding* c. Report any measures for improving the credibility of findings or verifying interpretations* | Yes |
|  | NO |
|  | Yes |
|  | NO |
|  | Yes |
|  | Yes |
| **Results** |  |
| a. Report sample size and characteristics of participants b. Support thematic findings with extracts, quotes, images, or observations c. Provide synthesis and interpretation | Yes |
|  | Yes |
|  | Yes |
| **Discussion** |  |
| a. Describe assumptions of the research and details of setting and context to illustrate transferability of findings b. Describe relationship of findings, or new theory developed in the study, to existing theory c. Report limitations | Yes |
|  | Yes |
|  | Yes |

*Elements with an asterisk may need to be elaborated in an appendix to avoid lengthening the manuscript.
